# Plasma Electrolytic Oxidation of Magnesium Alloy AZ31B in Electrolyte Containing Al_2_O_3_ Sol as Additives

**DOI:** 10.3390/ma11091618

**Published:** 2018-09-05

**Authors:** Xiaohua Tu, Chengping Miao, Yang Zhang, Yaling Xu, Jiayou Li

**Affiliations:** College of Biological and Chemical Engineering, Jiaxing University, Jiaxing 314001, China; miaochengping@mail.zjxu.edu.cn (C.M.); zhang5101021yang@mail.zjxu.edu.cn (Y.Z.); xuyaling@mail.zjxu.edu.cn (Y.X.); lijiayou@mail.zjxu.edu.cn (J.L.)

**Keywords:** magnesium alloy, plasma electrolytic oxidation, Al_2_O_3_ sol, corrosion resistance

## Abstract

Plasma electrolytic oxidation (PEO) coatings were produced on AZ31B magnesium alloys in alkaline electrolytes with the addition of various concentrations of Al_2_O_3_ sols. Effects of Al_2_O_3_ sol concentrations on the microstructure, phase composition, corrosion resistance and hardness of PEO coatings were evaluated by scanning electron microscopy (SEM), X-ray diffraction (XRD), microhardness testing and potentiodynamic polarization measurements, respectively. It was revealed that the Al_2_O_3_ sol mostly participated in the formation of the ceramic coating and transferred into the MgAl_2_O_4_ phase. With the increase of the Al_2_O_3_ sol concentration in the range of 0–6 vol%, the coating performance in terms of the microstructure, diffraction peak intensity of the MgAl_2_O_4_ phase, corrosion resistance and microhardness was improved. Further increase of Al_2_O_3_ sol addition did not generate better results. This indicated that 6 vol% might be the proper Al_2_O_3_ sol concentration for the formation of PEO coatings.

## 1. Introduction

Magnesium and its alloys, as structural and functional materials, have been increasingly attracting attention in a wide range of industries, e.g., aerospace, automotive communication devices and biodegradable implants [1,2,3]. Unlike the other light metal of aluminum, the spontaneous oxide film formed on the surface of the magnesium alloy cannot provide satisfactory protection from corrosion due to the decrease of about 20% in volume. Against this background, a variety of engineering options, including electroless plating, electropolymerization, chemical conversion, and anodization are available to improve the corrosion properties of magnesium alloys [4,5,6,7].

Plasma electrolytic oxidation (PEO), which is a promising and environmentally friendly surface treatment developed from conventional anodization, can provide a dense, thick, ceramic-like coating on the surface of the magnesium. PEO employs eco-friendly alkaline electrolytes and generates the oxide coating under high voltages by a number of electrochemical, electro-thermal and plasma-chemical reactions [3,8,9,10]. The performance of the PEO coating depends on many processing conditions, such as chemical composition of the electrolyte, the nature of the magnesium alloy and electric parameters [11,12,13,14,15,16,17]. The chemical composition of the electrolyte exerts a significant influence on the final properties of morphology features, coating composition, coating formation kinetics and the corrosion resistance [18,19,20,21,22,23]. In particular, in order to improve the functionality of PEO coatings, a recent approach has been to incorporate nanoparticles into the oxide coating layer, such as Al_2_O_3_, TiO_2_, SiO_2_, ZrO_2_, WC, TiN, Si_3_N_4_ and PTFE [24,25,26,27,28,29,30,31,32,33,34,35,36]. Among these nanoparticle additives, the Al_2_O_3_ nanoparticle is a very attractive option as it will not only penetrate into the coating, but also chemically transfer into the MgAl_2_O_4_ phase. The latter is considered beneficial for the anticorrosion performance [35,36].

Sol–gel materials exhibit great potential for use to improve the corrosion resistance of magnesium alloys, as it is possible to offer a highly adherent and chemically inert coating on the metallic surface. ZrO_2_ sol, CeO_2_ sol, SiO_2_ sol and Al_2_O_3_ sol have been utilized to fabricate the sol–gel coatings and all the corresponding metal oxide coatings have good chemical stability and provide effective protection for the magnesium substrate [37,38,39,40,41]. Compared with the study of the effect of nanoparticles on the properties of the magnesium alloys by the PEO process, the addition of sol–gel materials into the electrolyte has not attracted much attention [42,43,44]. In fact, sol–gel materials are considered to be promising additives for the PEO process, as these materials can be negatively charged in the alkaline electrolyte and also can likewise incorporate nanoparticles into the PEO coating matrix to improve the coating structure and anticorrosion performance [42,45]. In the present work, Al_2_O_3_ sol with various concentrations was successfully used to modify the NaOH–Na_2_SiO_3_ electrolyte. The surface morphologies, compositions, hardness and anticorrosion performance of the PEO coatings formed in the alkaline electrolyte with various concentrations of Al_2_O_3_ sol were evaluated.

## 2. Materials and Methods

Rectangular plates of AZ31B magnesium alloy with a size of 25 mm × 25 mm × 2 mm were cut from AZ31B plate (Hongdi Metal, Dongguan, China). Prior to PEO treatment, surfaces of all specimens were polished with successively finer grades of emery paper from 180 to 1000 grits. Then, the specimens were rinsed with distilled water and degreased with acetone.

The electrolyte composition used in the PEO process was based on NaOH (5 g/L) and Na_2_SiO_3_ (20 g/L). The coatings were obtained in the electrolyte with and without Al_2_O_3_ sol. The concentrations of Al_2_O_3_ sol added to the electrolyte were 0, 2, 4, 6, 8 and 10 vol%. The PEO process was performed by using a pulse electrical source (SOYI-30010M, Soyi Power, Shanghai, China). The electrical parameters were set as follows: frequency at 200 Hz, duty cycle at 10%, current density at 1.5 A/dm^2^. The PEO process time was 15 min and the temperature of the electrolyte was kept within the range 25–35 °C. The Al_2_O_3_ sol was prepared from aluminium isopropoxide, nitric acid and water with the molar ratio of 1:0.2:200. They were mixed and reacted under stirring for 24 h to prepare the Al_2_O_3_ sol at 85 °C.

Surface morphology of the PEO coatings was characterized by scanning electron microscopy (SEM, HITACHI, S–4800, Tokyo, Japan). The phase compositions of the coatings were analyzed by X-ray diffraction (XRD, DX-2600, Fangyuan Instrument, Dandong, China), using a Cu Kα radiation source. The surface roughness (Ra) of the coatings was measured using a stylus-type surface profilometer (TR200, Grows Precision Instrument, Shanghai, China). Each sample was measured five times and the average values are given. The film thickness was measured using an eddy current film thickness measurement gauge (MPO, Fisher, Sindelfingen, Germany). Coating hardness was evaluated by using an HXS–1000 microhardness tester (Milite Instrument, Nanjing, China).

Potentiodynamic polarization tests were carried out to evaluate the corrosion resistance of the coatings using CHI 842B electrochemical equipment (Chenhua, Shanghai, China) in 3.5 wt.% NaCl solution at 25 °C. During the measurements, a traditional three-electrode cell was used, with a large-area platinum sheet as counter electrode and a saturated calomel electrode (SCE) as reference electrode. Potentiodynamic polarization curves were obtained after 600 s immersion in the electrolyte, at a scan rate of 1 mV/s. The area of samples exposed to the electrolyte for the electrochemical tests was 1 cm^2^.

## 3. Results and Discussion

### 3.1. Plasma Electrolytic Oxidation Process

**Figure 1:**
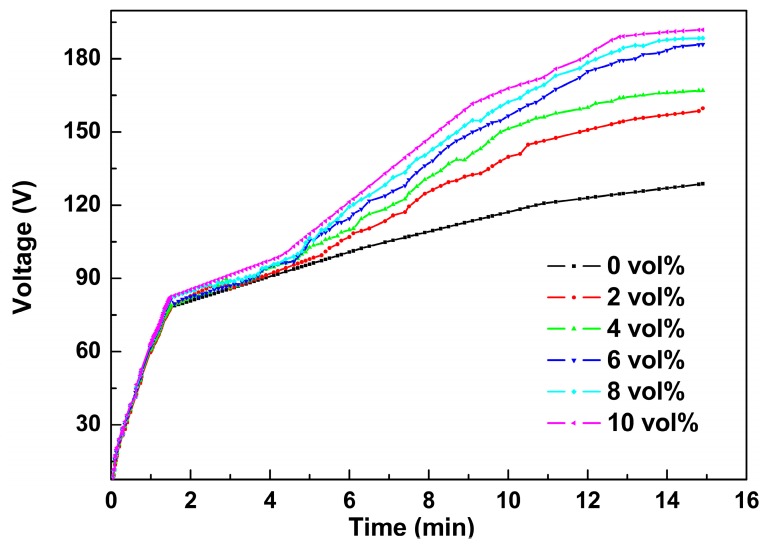
The potential change with time during the plasma electrolytic oxidation (PEO) process in alkaline electrolyte with different concentrations of Al_2_O_3_ sol.

Figure 1 shows the time-transient behaviors of voltage response of the magnesium alloy processed in the alkaline electrolytes with Al_2_O_3_ sol dosage from 0 to 10 vol%. Based on the alternations of slopes on the voltage–time curves, the PEO process can be divided into three stages. This phenomenon was similar to that proposed in the literature [8,19,22]. At Stage Ι (0–120 s), a small amount of gas bubbles is continuously released from the surface of the magnesium alloy substrate. The voltage increased quite sharply and almost linearly with the anodized time. It was estimated that a thin transparent coating was formed on the surface of the magnesium alloy. This passive coating can enhance the resistance of the PEO coating, which led to the rapid increase of the potential. When the voltage reached the “breakdown voltage”, one or two bright and small sparks appeared on the surface of the magnesium alloy. This indicated that the PEO process entered into Stage ΙΙ (about 120–720 s). Accordingly, the voltage still increased with respect to anodizing time, but the slopes of the curves decreased compared with those in Stage I. In this region, a number of white sparks in small sizes with shrill sound were observed on the surface of the anode. During Stage III (about 720–900 s), steady-state sparking occurred on the surface of the magnesium alloy. The voltages gradually reached the relatively steady value while the white sparks previously observed changed into separated orange ones. It is noteworthy that the breakdown voltages in the alkaline electrolytes with Al_2_O_3_ sols are higher than that without the additive. The reason for this may be that though a nonconductive additive, the Al_2_O_3_ sol particles may be adsorbed and trapped at the interface of alloy and electrolyte due to their negative charges and many hydroxyl groups [46,47,48]. This could be responsible for the enhancement of the surface resistance and thus have led to the increase of the breakdown voltages.

### 3.2. Coating Thickness of PEO Coatings

**Figure 2:**
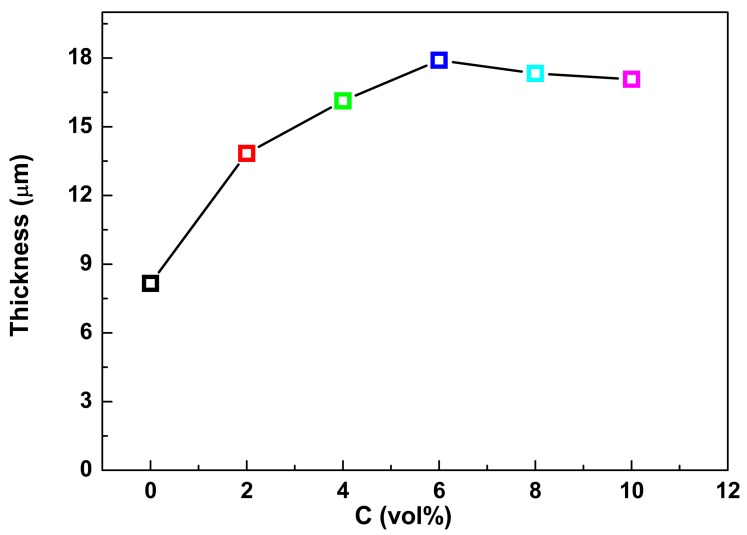
Effect of Al_2_O_3_ sol concentrations on thickness of PEO coatings.

The effect of Al_2_O_3_ sol concentration on the average thickness of PEO coatings formed on the magnesium alloy is shown in Figure 2. As seen from Figure 2, the addition of Al_2_O_3_ sols into the alkaline electrolyte for the PEO process resulted in a much thicker coating. In the region of 0–6 vol% Al_2_O_3_ sol, the coating thickness increases with increasing Al_2_O_3_ sol concentration and the thickest coating with the value of 17.9 μm is obtained with the addition of 6 vol% Al_2_O_3_ sol. The corresponding average growth rate of coating thickness was 1.19 μm/min, which was much slower than that reported in the literature [17]. This could be attributed to the much smaller current density in the PEO process compared with that reported elsewhere. However, with further increase of the Al_2_O_3_ sol concentration, the coating thickness drops slightly. These variations reveal that the additions of Al_2_O_3_ sol promoted the development of the PEO coating, while too much Al_2_O_3_ sol addition might have negative effects on the coating growth.

### 3.3. Coating Roughness of PEO Coatings

**Figure 3:**
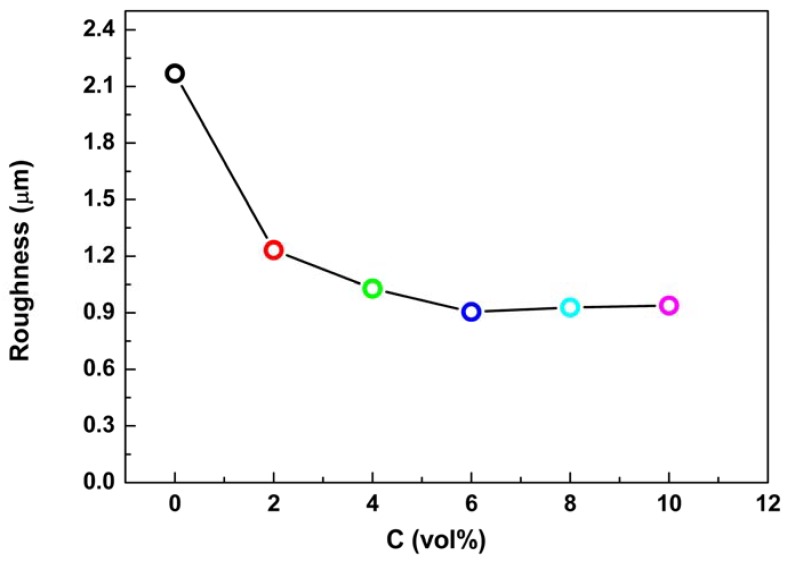
Effect of Al_2_O_3_ sol concentrations on roughness of PEO coatings.

Figure 3 presents the variation of average surface roughness of PEO coatings formed in the electrolyte with and without Al_2_O_3_ sol. Apparently, the surface roughness of the PEO coating formed in the electrolyte without Al_2_O_3_ sol was much higher than those of coatings formed in the electrolyte with various concentrations of Al_2_O_3_ sols. In the range of 0–6 vol% Al_2_O_3_ sol, the surface roughness dropped rapidly with the increase of the Al_2_O_3_ sol concentration. The roughness of the coating produced in the electrolyte with 6 vol% Al_2_O_3_ sol is about 0.9 μm. With further increase of the Al_2_O_3_ sol concentration, the surface roughness slightly increased. This indicated that the composition of the electrolyte, as well as the addition amount of Al_2_O_3_ sol, has a significant effect on the surface roughness of PEO coatings. The low surface roughness induced by Al_2_O_3_ sol might be beneficial to the corrosion resistance [49,50].

### 3.4. Morphological Characteristics of PEO Coatings

**Figure 4:**
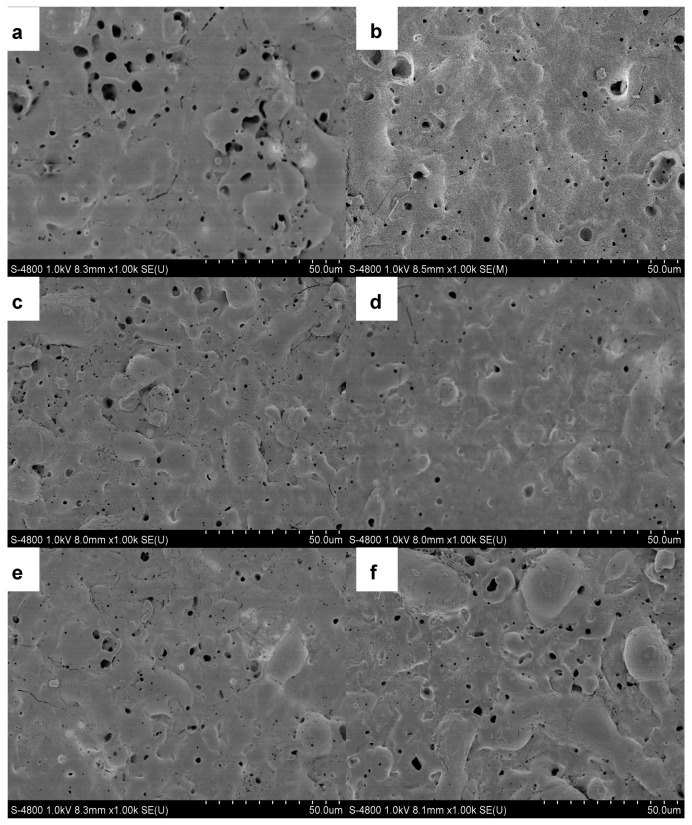
SEM images of PEO coatings in alkaline electrolyte with the addition of Al_2_O_3_ sol: (**a**) 0 vol%, (**b**) 2 vol%, (**c**) 4 vol%, (**d**) 6 vol%, (**e**) 8 vol%, (**f**) 10 vol%.

Figure 4 displays SEM images showing the morphologies of all PEO coatings formed in different compositions of electrolyte. Micropores, cracks and molten oxide could be observed on the surface of the PEO coatings in all samples, which were caused by the plasma discharge and gas release during the PEO process. However, the addition of Al_2_O_3_ sol into the alkaline electrolyte generated a significant change in the surface morphologies of PEO coatings. As shown in Figure 4a, the PEO coating formed in the alkaline electrolyte without Al_2_O_3_ sol had a rougher surface appearance. Many micropores with a diameter of 5–10 μm and several cracks could be found on the surface of the PEO coatings. This open structure of the porous and rough surface of the PEO coating could be easily penetrated by corrosive ions and thus cause corrosion. With the addition of Al_2_O_3_ sol into the electrolyte, the number and diameter of micropores decreased, as shown in Figure 4b–f. As can be seen in these micrographs, the surface of the coating formed in the electrolyte with 6 vol% Al_2_O_3_ sol was much more uniform compared with the others. Micropores, cracks and the molten oxide on the surface of the PEO coating will affect the surface roughness. The SEM image results of these PEO coatings are consistent with the surface roughness tests presented in Figure 3.

### 3.5. Phase Analysis of PEO Coatings

**Figure 5:**
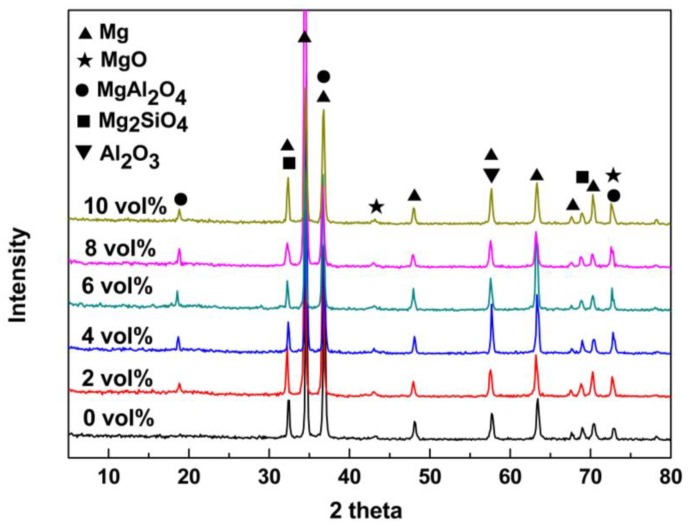
XRD patterns of the bare and PEO-coated magnesium alloys prepared with different concentrations of Al_2_O_3_ sol in electrolytes.

To understand the phase composition of the PEO coatings, XRD patterns are analyzed for the coated magnesium alloys prepared with different concentrations of Al_2_O_3_ sol in the electrolyte, as presented in Figure 5. The addition of Al_2_O_3_ sol to the electrolyte clearly had a great influence on the PEO coating composition. The PEO coating formed in the electrolyte without Al_2_O_3_ sol was mainly composed of MgO and Mg_2_SiO_4_. As the Al_2_O_3_ sol was added to the electrolyte, the MgAl_2_O_4_ phase with the diffraction peak value of 18.8° appeared in addition to the substrate peaks. As the concentration of Al_2_O_3_ sol increased from 2 vol% to 6 vol%, the intensity of the diffraction peak related to the MgAl_2_O_4_ phase increased significantly. With further increasing of the Al_2_O_3_ sol concentration, the intensity of the diffraction peak of the MgAl_2_O_4_ phase was less affected. This indicates that the Al_2_O_3_ sol participated in the formation of the PEO coating and transferred to the MgAl_2_O_4_ phase through some chemical reactions [35,36]. The formation mechanisms of PEO coatings with Al_2_O_3_ sol could be proposed as presented below in Equations (1)–(5) [3,20].

(1) $$\mathrm{Mg}\to {\mathrm{Mg}}^{2+}+2e^{-}\;(\mathrm{anodic}\;\mathrm{dissolution})$$

(2) $${\mathrm{Mg}}^{2+}+2{\mathrm{OH}}^{-}\to {\mathrm{MgO}+H}_{2}O$$

(3) $$\mathrm{Alumina}\;\mathrm{sol}\overset{\mathrm{PEO}}{⟶}{\mathrm{Al}}_{2}O_{3}$$

(4) $${\mathrm{Al}}_{2}O_{3}+\mathrm{MgO}\overset{\mathrm{PEO}}{\to }{\mathrm{MgAl}}_{2}O_{4}$$

(5) $${2\mathrm{Mg}}^{2+}+{\mathrm{SiO}}_{3}^{2-}+2{\mathrm{OH}}^{-}\overset{\mathrm{PEO}}{\to }{\mathrm{Mg}}_{2}{\mathrm{SiO}}_{4}+H_{2}O$$

### 3.6. Corrosion Behavior of PEO Coatings

**Figure 6:**
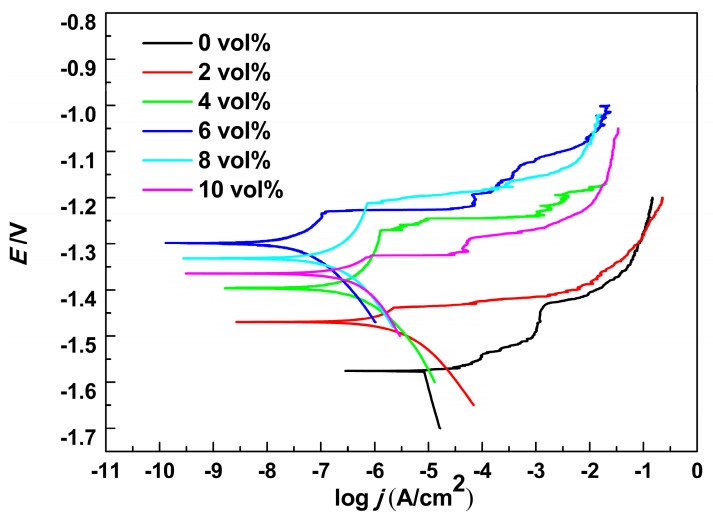
Potentiodynamic polarization behavior of PEO coatings formed in electrolytes with different concentrations of Al_2_O_3_ sol in 3.5 wt.% NaCl solution.

The anticorrosion performance of all PEO coating samples was evaluated by potentiodynamic polarization testing in 3.5 wt.% NaCl solution. The parameters, related to the corrosion properties such as corrosion potential (*E*_corr_), corrosion current density (*j*_corr_) and anodic (*β*_a_) and cathodic (*β*_c_) Tafel slopes are extracted directly from the potentiodynamic polarization curves displayed in Figure 6. The polarization resistances (*R*_p_) were calculated from the Equation

(6) $$R_{p}=\frac{\beta_{a}\beta_{b}}{2.303j_{\mathrm{corr}}\left(\beta_{a}+\beta_{b}\right)}$$

All corresponding parameter values are summarized in Table 1.

Corrosion potential (*E*_corr_), corrosion current density (*j*_corr_) and polarization resistances (*R*_p_) are often used to evaluated the anticorrosion performance of PEO coatings. A higher polarization resistance (*R*_p_), lower corrosion current density (*j*_corr_) and more positive corrosion potential (*E*_corr_) indicate a better corrosion resistance. As can been seen from Figure 6 and Table 1, the *E*_corr_, *j*_corr_ and *R*_p_ of the PEO coating formed without Al_2_O_3_ sol are −1.58 V, 8.52 × 10^−6^ A/cm^2^, and 1570 Ω·cm^2^, respectively. In terms of PEO coatings with various concentrations of Al_2_O_3_ sol, the *E*_corr_ shifted to the positive direction, *R*_p_ increased and *j*_corr_ decreased. This indicated that the addition of Al_2_O_3_ sol promoted the enhancement of the anticorrosion properties of the PEO coatings. This improvement of the anticorrosion properties of the PEO coating may be due to the increase of the coating thickness and the decrease of the coating roughness, which result in better anticorrosion properties, as mentioned above. In addition, the Al_2_O_3_ sol might be introduced into the micropores and the cracks during the PEO process, which would promote the inhibition of corrosive ion penetration into the magnesium alloy matrix. More importantly, the Al_2_O_3_ sol might be chemically transferred into the MgAl_2_O_4_ phase which can improve the anticorrosion performance [35,36]. It is noted that the corrosion current density of the PEO coating with 6 vol% Al_2_O_3_ sol reaches the minimum value of 5.18 × 10^−8^ A/cm^2^ and the associated corrosion potential is 1.30 V; also, the polarization resistance reaches the maximum value of 607,446 Ω·cm^2^. Based on the results of electrochemical parameters from Table 1, it was considered that the PEO coatings formed in the electrolyte with 6 vol% Al_2_O_3_ sol also showed good corrosion resistance compared with other PEO coatings of AZ31/AZ31B magnesium alloys in other reports [32,33,46,47]. In summary, 6 vol% is a proper concentration of Al_2_O_3_ sol to improve the anticorrosion property of a PEO coating. 

### 3.7. Microhardness of PEO Coatings

**Figure 7:**
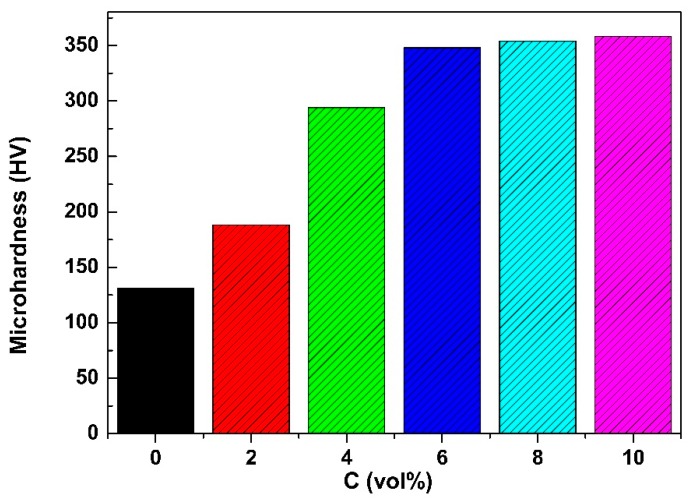
Effect of Al_2_O_3_ sol concentrations on hardness of PEO coatings.

The average microhardness of the PEO coatings formed in the electrolytes with various concentrations of Al_2_O_3_ sol is shown in Figure 7. The microhardness of the PEO coating without Al_2_O_3_ sol was 131 HV. The microhardness of all PEO coatings produced with Al_2_O_3_ sol was much higher than that of the coating produced without Al_2_O_3_ sol. Meanwhile, the microhardness of the PEO coatings increased significantly in the range of 0–6 vol% Al_2_O_3_ sol concentration and slightly changed with further increase of the Al_2_O_3_ sol. The PEO coating produced in the electrolyte with 6 vol% Al_2_O_3_ sol exhibits a microhardness of 348 HV, which is more than twice that of the PEO coating formed in the electrolyte without Al_2_O_3_ sol. The enhancement of the microhardness of the PEO coating is mainly due to the improvement of the microstructure and the increase of the MgAl_2_O_4_ phase, both of which promote the enhancement of the microhardness [36].

## 4. Conclusions

PEO coatings on magnesium alloy AZ31B were prepared in an alkaline electrolyte with various concentrations of Al_2_O_3_ sol. The surface morphology of the PEO coatings produced in the alkaline electrolytes with Al_2_O_3_ sol was more uniform with less structural imperfections than that of the coating produced in the electrolyte without Al_2_O_3_ sol. It was found that the Al_2_O_3_ sol mostly transferred into MgAl_2_O_4_ under certain chemical reactions, which also led to the better microhardness and anticorrosion performance. Moreover, under the same PEO conditions, the coating thickness and surface roughness were improved with the addition of Al_2_O_3_ sol in the alkaline electrolyte. However, when the Al_2_O_3_ sol exceeded 6 vol%, these trends of the effects of the addition of Al_2_O_3_ sol diminished. In sum, 6 vol% Al_2_O_3_ sol addition might be the better concentration for PEO coating formation for magnesium alloy AZ31B.

## Figures and Tables

**Table 1:** Electrochemical parameters related to potentiodynamic polarization curves for PEO coatings formed in electrolytes with different concentrations of Al_2_O_3_ sol.

Samples	*E*_corr_ (V)	*j*_corr_ (A/cm^2^)	*β*_a_ (V/Decade)	*β*_c_ (V/Decade)	*R*_p_ (Ω·cm^2^)
0 vol%	−1.58	8.52 × 10^−6^	0.031	0.406	1570
2 vol%	−1.47	2.53 × 10^−6^	0.022	0.104	3121
4 vol%	−1.40	5.61 × 10^−7^	0.313	0.134	72,720
6 vol%	−1.30	5.18 × 10^−8^	0.156	0.135	607,446
8 vol%	−1.33	3.29 × 10^−7^	0.271	0.198	151,195
10 vol%	−1.37	4.77 × 10^−7^	0.118	0.177	64,534

## References

[1] Gray X., Luan B. (2002). Protective coatings on magnesium and its alloys—A critical review. J. Alloys Compd..

[2] Chen X., Birbilis N., Abbott T. (2011). Review of corrosion-resistant conversion coatings for magnesium and its alloys. Corrosion.

[3] Song G., Shi Z., Song G. (2013). Anodization and corrosion of magnesium (Mg) alloys. Corrosion Prevention of Magnesium Alloys.

[4] Elsentriecy H.H., Azumi K., Konno H. (2007). Improvement in stannate chemical conversion coatings on AZ91 D magnesium alloy using the potentiostatic technique. Electrochim. Acta.

[5] Song Y., Shan D., Han E. (2007). Corrosion behaviors of electroless plating Ni–P coatings deposited on magnesium alloys in artificial sweat solution. Electrochim. Acta.

[6] Song G., Shi Z. (2014). Corrosion mechanism and evaluation of anodized magnesium alloys. Corros. Sci..

[7] Baloch A., Kannan M.B. (2017). Electropolymerisation of aniline on AZ91 magnesium alloy: The effect of coating electrolyte corrosiveness. Metals.

[8] Verdier S., Boinet M., Maximovitch S., Dalard F. (2005). Formation, structure and compostion of anodic films on AM60 magnesium alloy obtained by DC plasma anodizing. Corros. Sci..

[9] Zhang R. (2010). Film formation in the second step of micro-arc oxidation on magnesium alloy. Corros. Sci..

[10] Salman S., Okido M., Song G. (2013). Anodization of magnesium (Mg) alloys to improve corrosion resistance. Corrosion Prevention of Magnesium Alloys.

[11] Rama Krishna L., Sundararajan G. (2014). Aqueous corrosion behavior of micro arc oxidation (MAO)-coated magnesium alloys: A critical review. JOM.

[12] Li Z., Yuan Y., Jing X. (2014). Comparison of plasma electrolytic oxidation coatings on Mg-Li alloy formed in molybdate/silicate and aluminates/silicate composite electrolytes. Mater. Corros..

[13] Yagi S., Kuwabara K., Fukuta Y., Kubota K., Matsubara E. (2013). Formation of self-repairing anodized film on ACM522 magnesium alloy by plasma electrolytic oxidaition. Corros. Sci..

[14] Wu X., Su P., Jiang Z., Meng S. (2010). Influences of current density on tribological characteristics of ceramic coatings on ZK60 Mg alloy by plasma electrolytic oxidation. ACS Appl. Mater. Interfaces.

[15] Veys-Renaux D., Barchiche C., Rocca E. (2014). Corrosion behavior of AZ91 Mg alloy anodized by low-energy micro-arc oxidation: Effect of aluminates and silicates. Surf. Coat. Technol..

[16] Birss V., Xia S., Yue R., Rateick G. (2004). Characterization of oxide films formed on Mg-based WE43 alloy using AC/DC anodizaition in silicate solution. J. Electrochem. Soc..

[17] Arrabal R., Matykina E., Hashimoto T., Skeldon P., Thompson G. (2009). Characterization of AC PEO coatings on magnesium alloys. Surf. Coat. Technol..

[18] Hwang D., Kim Y., Park D., Yoo B., Shin D. (2009). Corrosion resistance of oxide layers formed on AZ91 Mg alloy in KMnO_4_ electrolyte by plasma electrolytic oxidation. Electrochim. Acta.

[19] Guo H., An M., Huo H., Xu S., Wu L. (2006). Microstructure characteristic of ceramic coatings fabricated on magnesium alloys by micro-arc oxidation in alkaline silicate solutions. Appl. Surf. Sci..

[20] Fukuda H., Matsumoto Y. (2004). Effects of Na_2_SiO_3_ on anodization of Mg-Al-Zn alloy in 3M KOH solution. Corros. Sci..

[21] Hsiao H., Tsung H., Tsai W. (2005). Anodization of AZ91D magnesium alloy in silicate-containing electrolytes. Surf. Coat. Technol..

[22] Wu H., Cheng Y., Li L., Chen Z., Wang H., Zhang Z. (2007). The anodization of ZK60 magnesium alloy in alkaline solution containing silicate and the corrosion properties of the anodized films. Appl. Surf. Sci..

[23] Lu J., He X., Li H., Song R. (2018). Microstructure and corrosion resistance of PEO coatings formed on KBM10 Mg alloy pretreated with Nd(NO_3_)_3_. Materials.

[24] Mandelli A., Bestetti M., Da Forno A., Lecis N., Trasatti S., Trueba M. (2011). A composite coating for corrosion protection of AM60B magnesium alloy. Surf. Coat. Technol..

[25] Zhang Y., Xu Y., Miao C., Tu X., Yu J., Li J. (2018). Effect of Tungsten Carbide Particles on the Characteristics of PEO Coatings Formed on AZ31B Magnesium Alloy in Alkaline Electrolyte. Int. J. Electrochem. Sci..

[26] Mashtalyar D., Gnedenkov S., Sinebryukhov S., Imshinetskiy I., Puz A. (2017). Plasma electrolytic oxidation of the magnesium alloy MA8 in electrolytes containing TiN nanoparticles. J. Mater. Sci. Technol..

[27] Lu X., Blawert C., Kainer K., Zheludkevich M. (2016). Investigation of the formation mechanisms of plasma electrolytic oxidation coatings on Mg alloy AM50 using particles. Electrochim. Acta.

[28] Lu X., Blawert C., Huang Y., Ovri H., Zheludkevich M., Kainer K. (2016). Plasma electrolytic oxidation coatings on Mg alloy with addition of SiO_2_ particles. Electrochim. Acta.

[29] Lou B., Lin Y., Tseng C., Lu Y., Duh J., Lee J. (2017). Plasma electrolytic oxidation coatings on AZ31 magnesium alloys with Si_3_N_4_ nanoparticle additives. Surf. Coat. Technol..

[30] Lee K., Shin K., Namgung S., Yoo B., Shin D. (2011). Electrochemical response of ZrO_2_-incorporated oxide layer on AZ91 Mg alloy processed by plasma electrolytic oxidation. Surf. Coat. Technol..

[31] Guo J., Wang L., Wang S., Liang J., Xue Q., Yan F. (2009). Preparation and performance of a novel multifunctional plasma electrolytic oxidation composite coating formed on magnesium alloy. J. Mater. Sci..

[32] White L., Koo Y., Yun Y., Sankar J. (2013). TiO_2_ deposition on AZ31 magnesium alloy using plasma electrolytic oxidation. J. Nanomater..

[33] Zhang D., Gou Y., Liu Y., Guo X. (2013). A composite anodizing coating containing superfine Al_2_O_3_ particles on AZ31 magnesium alloy. Surf. Coat. Technol..

[34] Arrabal R., Matykina E., Skeldon P. (2008). Incorporation of zirconia particles into coatings formed on magnesium by plasma electrolytic oxidation. J. Mater. Sci..

[35] Li X., Luan B.L. (2012). Discovery of Al_2_O_3_ particles incorporation mechanism in plasma electrolytic oxidation of AM60B magnesium alloy. Mater. Lett..

[36] Wang Y., Wei D., Yu J., Di S. (2014). Effects of Al_2_O_3_ nano-additive on performance of micro-arc oxidation coatings formed on AZ91D Mg alloy. J. Mater. Sci. Technol..

[37] Li Q., Chen B., Xu S., Gao H., Zhang L., Liu C. (2009). Structure and electrochemical behavior of sol-gel ZrO_2_ ceramic film on chemically pre-treated AZ91D magnesium alloy. J. Alloys Compd..

[38] Zhong X., Li Q., Hu J., Lu Y. (2008). Characterization and corrosion studies of ceria thin film based on fluorinated AZ91D magnesium alloy. Corros. Sci..

[39] Feil F., Furbeth W., Schutze M. (2008). Nanoparticle based inorganic coatings for corrosion protection of magnesium alloys. Surf. Eng..

[40] Zhong X., Li Q., Chen B., Wang J., Hu J., Hu W. (2009). Effect of sintering temperature on corrosion properties of sol-gel based Al_2_O_3_ coatings on pre-treated AZ91D magnesium alloy. Corros. Sci..

[41] Wang Z., Zeng R. (2010). Comparison in characterization of composite and sol-gel coating on AZ31 magnesium alloy. Trans. Nonferrous Met. Soc. China.

[42] Zhu L., Li Y., Li W. (2008). Influence of silica sol particles behavior on the magnesium anodizing process with different anions addition. Surf. Coat. Technol..

[43] Tang M., Liu H., Li W., Zhu L. (2011). Effect of zirconia sol in electrolyte on the characteristics of microarc oxidation coating on AZ91D magnesium. Mater. Lett..

[44] Guo Y., Wang G., Dong G., Zhang L., Zhang M. (2008). Corrosion resistance of anodized AZ31 Mg alloy in borate solution containing titania sol. J. Alloys Compd..

[45] Li Z., Jing X., Yuan Y., Zhang M. (2012). Composite coatings on a Mg-Li alloy prepared by combined plasma electrolytic oxidation an sol-gel techniques. Corros. Sci..

[46] Guo X., An M., Yang P., Su C. (2009). Effects of benzotriazole on anodized film formed on AZ31B magnesium alloy in environmental-friendly electrolyte. J. Alloys Compd..

[47] Zhu F., Wang J., Li S., Zhang J. (2012). Preparation and characterization of anodic films on AZ31B Mg alloy formed in the silicate electrolyte with ethylene glycol oligomers as additives. Appl. Surf. Sci..

[48] Tu X., Chen L., Wu J. (2013). Effect of glucose on properties of anodizing film on AZ31B magnesium alloy. Chin. J. Nonferrous Met..

[49] Yoo B., Shin K., Hwang D., Lee D., Shin D. (2010). Effect of surface roughness on leakage current and corrosion resistance of oxide layer on AZ91 Mg alloy prepared by plasma electrolytic oxidation. Appl. Surf. Sci..

[50] Kamil M., Kaseem M., Lee Y., Ko Y. (2017). Microstructure characteristics of oxide layer formed by plasma electrolytic oxidation: Nanocrystalline and amorphous structure. J. Alloys Compd..

